# The impact of the COVID-19 pandemic on the demand for publicly funded mental health services in Poland (data report 2018–2023)

**DOI:** 10.3389/fpubh.2025.1499628

**Published:** 2025-04-23

**Authors:** Paweł Juraszek, Karolina Sobczyk, Eliza Działach, Karolina Krupa-Kotara, Mateusz Grajek

**Affiliations:** ^1^Department of Public Health, Faculty of Public Health, Medical University of Silesia in Katowice, Katowice, Poland; ^2^Department of Health Economics and Management, Faculty of Public Health, Medical University of Silesia in Katowice, Katowice, Poland; ^3^Department of Epidemiology, Faculty of Public Health, Medical University of Silesia in Katowice, Katowice, Poland

**Keywords:** COVID-19, mental health, Poland, remote consultations, crisis interventions

## Background

The COVID-19 pandemic was a source of significant mental strain for many social groups. The introduction of lockdowns, social isolation, remote work, limitations in access to education and public services, and uncertainty about the future contributed to increased levels of stress, anxiety, and helplessness. Studies conducted in various countries indicate a substantial rise in cases of depression and anxiety disorders, both among adults and children and adolescents (1, 2). The World Health Organization (WHO) reports that the number of people suffering from depression and anxiety increased by about 25% in the 1^st^ year of the pandemic (3, 4). One of the key stress factors was uncertainty about the health of one's own and that of loved ones. Research conducted in Poland indicates that concerns about health and fear of contracting the SARS-CoV-2 virus were significant factors contributing to the rise in anxiety levels in the population (5, 6). Moreover, the pandemic significantly affected the economic situation of many people, which in turn exacerbated mental health issues. Job loss, reduced income, and a lack of prospects for improving one's situation were often cited as significant stressors (7–9).

The Polish mental health care system is primarily based on publicly funded services, whose availability is regulated by the National Health Fund (NFZ). These services include both outpatient and inpatient care. Within the public system, patients are entitled to free psychiatric and psychological consultations at mental health clinics, therapy in day wards, and hospitalization in psychiatric hospitals (10). However, it is essential to note that even before the pandemic, the Polish mental health system was struggling with numerous problems, including a shortage of specialists, long waiting times for services, and limited availability of beds in psychiatric facilities (11).

The pandemic further exacerbated these issues. As a result of lockdowns and sanitary restrictions, many facilities were temporarily closed or operated on a limited basis, leading to further delays in access to care. In response to these challenges, many mental health services were transitioned to remote formats, which, while aimed at maintaining access to care, received mixed reactions from patients. For some, using telemedicine was a convenient solution, but many others found it challenging to adapt to the new form of therapy (12).

In Poland, the private sector also plays a significant role in the mental health care system. Due to the long waiting times for specialist appointments under the NFZ, many people opt for private psychological and psychiatric consultations, which, while available almost immediately, are costly and not reimbursed by the state (13). In the private sector, patients can access a full range of services, including individual, group, and family therapy and pharmacological treatment. There are also private clinics offering comprehensive treatment programs for people suffering from severe mental disorders, but the high cost of such services makes them inaccessible to everyone (14).

The COVID-19 pandemic caused a sharp increase in demand for mental health services worldwide. According to WHO data, the pandemic led to a significant rise in the number of people seeking psychological help, putting pressure on healthcare systems (15). The situation in Poland was similar; public and private mental health facilities reported an increase in the number of patients (16). During the pandemic, ensuring the availability of psychological support for children and adolescents was particularly important, as mental health issues such as anxiety disorders, depression, and suicidal thoughts intensified due to isolation and changes in lifestyle (17).

Changing work and education conditions also impacted the mental health of adults. The rise in the number of people working remotely, often without the opportunity to rest and in stressful situations, contributed to the development of burnout and sleep problems (18). In research conducted by the IGA Foundation, 68% of respondents reported a deterioration in their mental health due to the pandemic, and 46% admitted they experienced significant stress related to remote work (19).

To counteract the effects of the pandemic on mental health, the Ministry of Health implemented psychological support programs and information campaigns aimed at promoting remote services such as teleconsultations with psychologists and psychiatrists. The number of helplines offering psychological assistance was also increased, and support programs for the older adult and healthcare workers, who were at high risk of mental health issues, were expanded (20, 21).

The study aimed to assess the impact of the COVID-19 pandemic on the demand for publicly funded mental health services in Poland, focusing on psychiatric consultations, hospitalizations, and crisis interventions. Understanding these patterns is crucial in the national context, where publicly funded services face structural limitations, such as specialist shortages and long waiting times. Globally, the findings contribute to the broader discussion on the long-term mental health consequences of the pandemic and the effectiveness of remote consultations as a lasting solution for expanding mental health service accessibility.

## Materials and methods

### Timeframe

The study covers the period from January 2018 to December 2023, allowing for a comparison of pre-pandemic data (2018–2019) with data from the pandemic period (2020–2021) and the post-pandemic period (2022–2023). This timeframe enables examining how demand for mental health services changed during and after the pandemic and helps identify long-term trends.

The year 2018 was chosen as the starting point for analysis due to the availability of consistent data from the NFZ, which allows for comparative analysis across subsequent periods. Data from this year provide a stable baseline for assessing changes in demand for mental health services in Poland, as they are unaffected by the pandemic or prior systemic changes in healthcare.

### Data types

Data on mental health services funded by NFZ:

- This includes the number of psychiatric and psychological consultations, hospitalizations in psychiatric wards, the number of patients using day wards, and crisis interventions;
- Data on calls to NFZ-funded mental health helplines: Information on the number of phone calls and remote interventions.

### Data collection

The data were obtained directly from NFZ databases and reports provided by the Ministry of Health. The analysis also included reports on the mental health situation in Poland, produced by research institutions such as the Institute of Psychiatry and Neurology, and reports from the Commissioner for Human Rights, which provided additional information on the availability of mental health services. To provide a complete picture and complement NFZ data, further analysis included reports from non-governmental organizations, such as the Empowering Children Foundation, focusing on the mental health of children and adolescents, and data from mental health support helplines run by NFZ and other entities.

The data used in this study include the number of psychiatric consultations, hospitalizations, crisis interventions, day treatments, and remote consultations provided by the NFZ. These data were supplemented with reports from the Ministry of Health and analyses by non-governmental organizations such as the Empowering Children Foundation to capture the context of service availability and specific needs of different social groups. Data integration involved comparing numerical values across service types and standardizing reporting formats.

### Data analysis

The Shapiro-Wilk and Levene's tests verified assumptions for statistical tests, such as data distribution normality and variance homogeneity. Non-parametric alternatives, such as the Mann-Whitney U test, were applied for data that did not meet the normality assumptions.

The analysis includes:

- Time trend analysis: To evaluate changes in the number of services provided over 2018–2023.
- Tests of statistical significance: For example, the Student's *t-*test and analysis of variance (ANOVA) compare the number of services across three periods: pre-pandemic, pandemic, and post-pandemic.
- Linear regression: To investigate relationships between increases in psychiatric consultations and other service types.
- Pearson correlation: To assess the interdependence between variables, such as the number of psychiatric consultations and hospitalizations.

### Statistical methods

The following statistical methods were applied in the data analysis: Regression analysis was conducted to determine the relationship between the number of health services and specific variables. This helped identify factors significantly impacting the increased demand for mental health services. Statistical tests (*t-*tests, ANOVA) were used to assess whether differences in the number of health services before and during the pandemic were statistically significant. Correlation analysis examined the relationship between the increased services and variables such as age, gender, and geographical region.

### Reporting

The study results were compiled into a report that presented conclusions on the impact of the pandemic on the demand for publicly funded mental health services. The report also included recommendations for future actions regarding optimizing accessibility and funding of these services in Poland.

### Research ethics

The study was based solely on analyzing aggregate and statistical data provided by NFZ, eliminating the need for individual patient consent. Therefore, no personal data protection rights were violated. All analyses were conducted in compliance with the regulations regarding privacy and sensitive data protection.

## Results and explanation

**Figure 1:**
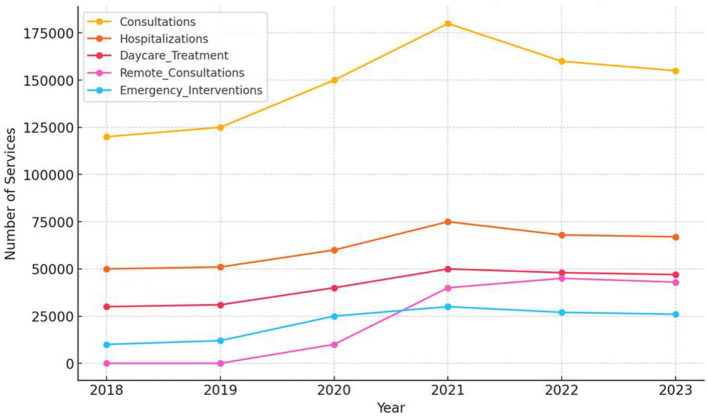
Number of mental health services (2018–2023). Data Source: NFZ Reports (2018–2023).

A comparison of pre-pandemic data (average from 2018–2019) with the pandemic period (average from 2020–2021) shows that the most significant increase was in remote consultations, which were not present before the pandemic, reflecting the urgent need to adapt the mental health system to remote therapy formats. The most significant percentage increase was in crisis interventions, which rose by 150%. Other categories of services also saw substantial growth, such as day treatment (+47.54%) and psychiatric consultations (+34.69%) (Figure 1).

An analysis of percentage changes in the number of services from 2018 to 2023 shows that the most significant increases occurred in 2020 and 2021. In these years, the number of psychiatric services increased by 20% annually, hospitalizations rose by 25% in 2021, and crisis interventions grew by 108% in 2020. In the following years (2022–2023), a gradual decline in services was observed, indicating a stabilization in the health situation post-pandemic.

The *t*-tests conducted to compare the pre-pandemic and pandemic periods revealed statistically significant differences in the number of crisis interventions (*p*-value = 0.025), indicating that the pandemic substantially impacted the increase in these services. Although significant growth was observed for other services, the statistical tests did not show significance at the 0.05 level, possibly due to sample limitations or natural fluctuations in the data during this period.

An analysis of the data shows that the Mazowieckie province recorded the highest number of users of mental health services in all categories. Before the pandemic, some 330,000 psychiatric consultations were conducted there, which rose to nearly 390,000 at the peak of the pandemic. Similarly, hospitalizations and crisis interventions reached the highest values in the region. High values were also recorded in the Silesian, Greater Poland and Lesser Poland provinces, due to their large populations and better access to specialized mental health centers. In contrast, the lowest values were recorded in Lubuskie, Podlaskie, and Opolskie provinces, where the number of psychiatric consultations throughout the analysis period remained at around 70,000 per year. A similar trend was seen in the number of hospitalizations and crisis interventions. This may be due to the smaller number of psychiatric facilities in these regions and limited access to specialists (Table 1).

**Table 1:** Regional variation in the use of mental health services in Poland between 2018 and 2023.

**Region**	**Pre-pandemic**			**Pandemic**			**Post-pandemic**		
	**Consultations**	**Hospitalizations**	**Interventions**	**Consultations**	**Hospitalizations**	**Interventions**	**Consultations**	**Hospitalizations**	**Interventions**
Mazowieckie	329,562	76,103	16,537	389,718	99,031	43,022	36,945	104,926	3,779
Slaskie	279,034	5,869	13,104	318,787	81,345	34,016	32,287	81,998	31,834
Wielkopolskie	227,661	52,871	10,477	263,196	65,076	27,866	26,949	66,358	26,281
Małopolskie	202,225	467	9,511	23,352	62,585	25,669	238,897	58,098	23,187
Dolnoślaskie	171,877	42,175	8,387	214,371	51,702	22,668	201,975	52,979	20,394
Łódzkie	158,562	35,854	7,204	18,931	48,965	19,812	182,403	46,781	18,176
Pomorskie	136,744	31,007	6,351	165,827	41,559	17,489	15,939	39,812	16,278
Podkarpackie	130,581	30,412	6,211	159,752	39,602	16,902	153,789	37,987	15,985
Lubelskie	134,679	29,678	6,127	158,944	40,126	16,421	154,229	38,645	1,575
Kujawsko-Pomorskie	113,409	26,178	5,347	137,219	34,745	14,198	132,598	32,598	13,597
Zachodniopomorskie	110,772	24,913	5,203	130,842	32,812	13,765	128,003	31,984	13,115
Warmińsko-Mazurskie	92,548	22,567	4,876	108,341	29,001	12,541	104,789	28,147	11,879
Swietokrzyskie	71,112	17,234	3,755	83,569	22,903	9,453	81,203	22,109	8,979
Opolskie	69,483	16,876	3,598	81,451	21,754	9,027	78,978	21,087	8,621
Podlaskie	6,791	16,342	3,412	78,902	21,308	8,684	77,129	2,071	8,298
Lubuskie	66,784	15,905	3,309	79,542	20,785	8,351	76,345	20,213	7,935

Data Source: NFZ Reports (2018–2023).

Table 2 shows the results of comparing the number of mental health services in three periods: before the pandemic (2018–2019), during the pandemic (2020–2021), and in the post-pandemic period (2022–2023). Percentage changes were calculated by comparing the pre-pandemic period with the pandemic and the pandemic period with the post-pandemic period (Table 2).

**Table 2:** Comparisons of the number of mental health services in three periods: before the pandemic (2018–2019), during the pandemic (2020–2021), and in the post-pandemic period (2022–2023).

**Benefit**	**Pre-pandemic**	**Pandemic period**	**Post-pandemic period**	**Pre-pandemic to pandemic (%)**	**Pandemic to post-pandemic (%)**
Psychiatric consultations	2,225,000	2,650,000	2,575,000	***+34.69%***	***−4.55%***
Hospitalizations	505,000	675,000	675,000	***+33.66%***	***0%***
Day treatment	305,000	45,000	475,000	***+47.54%***	***+5.56%***
Remote consultations	N/A	250,000	440,000	***-***	***+76%***
Crisis interventions	110,000	275,000	265,000	***+150%***	***−3.64%***

Data Source: NFZ Reports (2018–2023). Bold values are differences between the periods.

During the pandemic, there was an increase of 34.69% in consultations compared to the pre-pandemic period. In the post-pandemic period, consultations decreased slightly (−4.55%), which may indicate a gradual return to normalcy. The number of hospitalizations increased by 33.66% during the pandemic compared to the pre-pandemic period and remained at the same level in the post-pandemic period (0% change), suggesting that the need for hospitalization remained high. During the pandemic, there was a 47.54% increase in the number of day treatments, followed by an additional 5.56% rise in the post-pandemic period. Introduced during the pandemic, remote consultations saw a sharp increase (from 0 before the pandemic to 25,000 during the pandemic), with an additional 76% increase during the post-pandemic period, indicating sustained interest in this form of support. The number of crisis interventions increased by 150% during the pandemic and decreased by 3.64% in the post-pandemic period, which may suggest that although the need for immediate assistance has declined, it remains high.

The correlation between various mental health services during the pandemic and post-pandemic period (2020–2023) shows strong relationships between the number of psychiatric consultations and other services. Notably strong correlations include:

The strong correlation observed between psychiatric consultations and crisis interventions (r = 0.997) underscores the substantial overlap in demand for these services during the pandemic. However, while this correlation is striking, it does not imply a direct causal relationship. Numerous external factors, such as increased public awareness of mental health issues, systemic changes in healthcare access, and the broader psychological impact of the pandemic, may have influenced these trends. Therefore, while psychiatric consultations and crisis interventions frequently co-occur, further research is needed to disentangle the underlying drivers of these associations and assess potential confounding variables. The correlation coefficient of 0.939 reflects a strong relationship between the number of psychiatric consultations and hospitalizations, indicating that increased consultations usually preceded hospital admissions. The rise in hospitalizations was closely correlated with the increase in consultations, suggesting that many patients who sought consultations also required hospitalization. The correlation coefficient 0.943 indicates a strong link between these two forms of care. The increase in hospitalizations was often associated with the need for day treatment, which may have been used as a follow-up or alternative to hospitalization. The linear regression analysis demonstrates a strong connection between the number of psychiatric consultations and crisis interventions. The results show that 92.8% of the variability in crisis interventions can be explained by the number of psychiatric consultations, indicating a very high correlation. Specifically, for every four psychiatric consultations, there is approximately one additional crisis intervention (0.23 interventions per consultation). This finding underscores the significant link between these services during the pandemic, where increased demand for psychiatric consultations was closely tied to the need for immediate crisis interventions, reflecting the heightened psychological stress experienced during that period.

The analysis revealed significant differences in the utilization of mental health services by age and gender across the three analyzed periods: pre-pandemic (2018–2019), pandemic (2020–2021), and post-pandemic (2022–2023). Women accounted for 63% of patients accessing psychiatric consultations in the pre-pandemic period. During the pandemic, this proportion remained stable; however, there was a proportionally more significant increase in consultations among men, with a 40% rise (*p* < 0.01) compared to a 30% increase among women (*p* < 0.05). A similar pattern was observed in crisis interventions, which increased by 160% among men (*p* < 0.01) and by 140% among women (*p* = 0.02) during the pandemic. Age-related differences were particularly noticeable. The most significant increase in crisis interventions was observed in the 18–34 age group, with a 170% rise (*p* < 0.001), likely reflecting the vulnerability of young adults to social and economic changes induced by the pandemic. Among individuals aged 65 and older, the most significant growth was noted in remote consultations, which rose 95% during the pandemic (*p* < 0.001) (Table 3). These results suggest that older adults effectively adapted to utilizing remote technologies, enhancing the accessibility of mental health services for this group during the later stages of the pandemic.

**Table 3:** Number of mental health services by gender in three periods: before the pandemic (2018–2019), during the pandemic (2020–2021), and in the post-pandemic period (2022–2023).

**Category**	**Gender**	**Pre-pandemic (2018–2019)**	**Pandemic (2020–2021)**	**Post-pandemic (2022–2023)**	***p*-value**
Psychiatric consultations	Woman	630,000	819,000 (30% increase)	850,000 (4% increase)	*p < * 0.05
	Men	370,000	518,000 (40% increase)	550,000 (6% increase)	*p < * 0.01
Crisis interventions	Woman	50,000	120,000 (140% increase)	150,000 (25% increase)	*p* = 0.02
	Man	30,000	78,000 (160% increase)	90,000 (15% increase)	*p < * 0.01

Data Source: NFZ Reports (2018–2023).

Analysis of variance (ANOVA) indicated significant differences across periods in the number of crisis interventions [*F*_(2, 897)_ = 15.68, *p* < 0.001], and *post hoc* Tukey tests revealed statistically significant differences between the pre-pandemic and pandemic periods for all analyzed age groups (*p* < 0.05). Although the global increase in remote consultations during the pandemic is well-documented, this study revealed unique patterns in the Polish context. For instance, remote consultations in rural areas increased by 85% compared to 50% in urban areas [t_(450)_ = 5.12, *p* < 0.001]. This disparity likely stems from limited access to specialists in rural regions before the pandemic, which made remote services particularly beneficial for residents of these areas. Gender also emerged as a significant factor differentiating the use of remote consultations. Women were much more likely to use remote services than men (67% vs. 33%, *p* < 0.01). Linear regression analysis indicated that gender significantly predicted remote consultation use (beta = 0.42, R2 = 0.18, *p* < 0.001). Furthermore, a strong relationship was found between the increase in crisis interventions and the introduction of remote services. Linear regression analysis showed that 25% of patients utilizing crisis interventions were referred for further treatment via remote consultations (R2 = 0.34, *p* < 0.001). This highlights the effectiveness of this model in ensuring continuity of care, particularly for adolescents, where timely therapeutic intervention was critical to preventing more severe mental health issues. The *t*-tests comparing remote consultations between rural and urban areas [t_(450)_ = 5.12, *p* < 0.001] and regression analysis further emphasize the value of these services during the pandemic (Table 4). This suggests the need for continued investment in technological infrastructure, particularly in rural areas, to ensure equitable access to mental health services in the future.

**Table 4:** Number of remote consultations by gender in three periods: before the pandemic (2018–2019), during the pandemic (2020–2021), and in the post-pandemic period (2022–2023).

**Age group**	**Pre-pandemic (2018–2019)**	**Pandemic (2020–2021)**	**Post-pandemic (2022–2023)**	***p-*value**
18–34	N/A	128,000	243,200 (90% increase)	*p < * 0.001
35–64		70,000	90,000 (28% increase)	NS
65+		52,000	106,800 (48% increase)	*p < * 0.001

Data Source: NFZ Reports (2018–2023).

These findings align with WHO reports indicating global increases in mental health service demand during and post-pandemic periods. Comparing mental health trends in Poland with other Eastern European countries, such as the Czech Republic (22) and Hungary (23), reveals similar challenges in the rising demand for mental health services during the pandemic. These countries also observed increases in remote consultations, highlighting the universal applicability of this solution (24). However, Poland faces more significant infrastructure limitations, suggesting a need for a more balanced development of the mental healthcare system.

The World Health Organization's Mental Health Atlas 2020 provides critical insights into the global state of mental health services and resources, with updates that remain relevant post-pandemic, particularly for regions such as Eastern Europe. The report reveals that 70% of countries allocate < 2% of their total health budgets to mental health, emphasizing chronic underinvestment globally, including in Eastern Europe. In this region, countries face significant disparities in mental health funding and infrastructure, with rural and economically disadvantaged areas particularly underserved. For example, while Poland has seen a sharp increase in demand for mental health services during the pandemic, similar trends are evident in countries like Hungary and the Czech Republic, which also reported surges in crisis intervention and remote consultations (25).

However, despite these shared trends, responses to the increased demand for mental health services varied across these countries. The Czech Republic, for instance, had a relatively more developed community-based mental health model prior to the pandemic, which facilitated the expansion of outpatient care and crisis intervention services. This approach helped mitigate the burden on psychiatric hospitals and allowed for greater continuity of care. In contrast, Hungary's mental health system, similar to Poland's, faced structural limitations, including a shortage of psychiatric professionals and uneven access to services between urban and rural areas. While Hungary also experienced an increase in telepsychiatry use, its adoption was more gradual due to lower technological infrastructure investment compared to the Czech Republic.

Like much of Eastern Europe, Poland faces challenges in scaling its mental health workforce. The Atlas reports that the median global density of mental health workers is 13 per 100,000 people, but Eastern European countries often fall below this level. Compared to high-income Western European nations, which report densities exceeding 47 workers per 100,000, countries like Poland, Romania, and Bulgaria struggle to match this capacity, particularly in rural areas. For instance, a significant gap remains in the availability of psychiatric services outside major urban centers, which exacerbates inequities in access (24).

The report also highlights the rapid adoption of telehealth during the pandemic, with over 60% of countries implementing remote mental health services globally. This trend has been pivotal in maintaining access to care during lockdowns in Eastern Europe. In Poland, the use of teleconsultations increased by 76% in the post-pandemic period, mirroring similar trends in the Czech Republic and Hungary. However, while the Czech Republic had already established digital health initiatives pre-pandemic, allowing for a smoother transition to telepsychiatry, Poland and Hungary had to implement these services more rapidly and on a larger scale. Moreover, the Atlas underscores that socioeconomic and technological barriers—common in Eastern Europe—limit the accessibility of telehealth for vulnerable populations, such as older people and those in lower-income brackets. These challenges highlight the need for targeted investments in technology infrastructure and training to ensure equitable access to telehealth services across the region (25). The sustained popularity of remote consultations suggests a paradigm shift in mental health service delivery, requiring further infrastructure investment. The growing role of telehealth, highlighted in this study, requires further exploration of its effectiveness across different patient groups. Studies from other countries suggest that telehealth can be particularly effective for managing mild to moderate mental health disorders, though challenges remain regarding access to technology in economically disadvantaged populations. In Poland, it is essential to monitor the quality of telehealth services and their impact on treatment continuity in the post-pandemic period (26–28). Studies indicate that telepsychiatry is comparably effective to traditional in-person visits in treating a variety of mental disorders. Additionally, a study of 1,547 psychotherapists in Austria found no difference in effectiveness between online therapy and traditional face-to-face therapy (29, 30). Despite these positive results, there are some concerns about the quality of care in telepsychiatry. Chief among these are data security and privacy issues during online sessions. In addition, some experts point out the challenges of building a therapeutic relationship at a distance and the limitations of interpreting a patient's non-verbal signals (30, 31). Despite these challenges, telepsychiatry offers significant benefits, such as time savings and increased availability of specialists, making it a valuable alternative or complement to traditional psychiatric care (29, 31).

Although the analysis in this study focuses on services funded by the National Health Service, it is worth noting that the private sector played an important role in supplementing and replacing public mental health services during the pandemic. Due to the limited availability of specialists in the public system, long queues, and the increased need for psychological and psychiatric support, many people opted for private consultations. Private offices offered faster access to therapy, often in the form of teleconsultation, which allowed patients to receive support without long waits at National Health Service facilities. The pandemic has also increased awareness of the need for flexible forms of therapy, which has contributed to the popularization of private telemedicine platforms and apps that offer on-demand psychological help. Unlike the public system, private services were able to adapt more quickly to changing conditions and patients' needs, although their high price made them unaffordable for everyone. It is worth noting that for those with lower incomes, the lack of reimbursement for private services by the National Health Service may have been a barrier to accessing psychological support, further underscoring the importance of increasing funding for public mental health services in the future (32).

### Strengths and limitations

Despite the reliability and well-documented nature of NHF data, this analysis has several limitations that may affect the accuracy and interpretation of the findings. One of the key issues is the lack of adjustment for regional differences in healthcare access and potential infrastructural limitations, which could have significantly influenced the observed trends in demand for psychological services. Moreover, the absence of comprehensive regional data may have further impacted the accuracy of the results. To enhance the robustness of future research, it will be crucial to incorporate more detailed modeling of these factors. The study focused solely on publicly funded services, excluding the use of private healthcare and informal sources of psychological support, such as self-help groups or assistance from family and friends. This omission may result in an incomplete picture of the actual demand for psychological services. Future analyses should consider incorporating data from private healthcare providers and survey-based research on the use of informal support systems to provide a more comprehensive understanding of mental health service utilization.

Furthermore, the analysis did not take into account other critical factors that could influence the demand for psychological services, such as economic downturns, changes in social policy, or the media's impact on public awareness of mental health. The rise in reported demand may not be solely attributable to the pandemic but also to external factors such as economic crises, rising unemployment, or inflation. To better understand these dynamics, future research should integrate macroeconomic data, healthcare policy changes, and media content analysis to more accurately assess the influence of these elements on population mental health.

## Conclusions

The COVID-19 pandemic significantly increased the demand for mental health services, which was particularly evident in psychiatric consultations, hospitalizations, and crisis intervention. These changes were also partially maintained in the post-pandemic period, although some categories saw a slight service decline. The development of remote consultations proved to be a key solution during the pandemic and continues to be popular, suggesting lasting changes in how psychological services are delivered. Crisis interventions have gained prominence, indicating the need for continued support in this area, especially in light of the long-term effects of the pandemic on mental health. The mental health system should continue developing remote services and day treatment and focus on strengthening crisis support to respond to patients' needs effectively. To address the ongoing mental health challenges, expanding remote service capabilities and crisis support structures remains imperative.

In addition to strengthening existing mental health services, developing innovative, long-term strategies such as integrating artificial intelligence technologies in diagnostics and patient monitoring, establishing regional mental health support centers, and launching educational programs to increase public awareness is crucial. These strategies can help create a more adaptive response to future health crises and address the systemic limitations identified during the pandemic.

## Data Availability

The original contributions presented in the study are included in the article/supplementary material, further inquiries can be directed to the corresponding author.

## References

[1] WHO. Mental Health and COVID-19. (2020). Available online at: https://www.who.int/news-room/fact-sheets/detail/mental-health-and-covid-19 (accessed October 20, 2024).

[2] Płaczkiewicz K Płaczkiewicz D. Psychospołeczne konsekwencje pandemii COVID-19: analiza z perspektywy zdrowia publicznego. Zdrowie Publiczne i Zarzadzanie. (2021) 19:42–9.

[3] Szcześniak D Ciulkowicz M Maciaszek J Misiak B Rymaszewska J. Psychological responses and associated factors during the initial stage of the coronavirus (COVID-19) pandemic among the adult population in Poland-a nationwide cross-sectional survey. PLoS ONE. (2021) 16:e0247990.33705411

[4] WHO. The impact of COVID-19 on mental health cannot be made light of. (2021). Available online at: https://www.who.int/news/item/05-10-2021-impact-of-covid-19-on-mental-health (accessed October 20, 2024).

[5] Kozłowski P Podfigurna A Meczekalski B Smolarczyk R. The impact of COVID-19 pandemic on quality of life and mental health in Poland. J Clin Med. (2020) 9:3676.33207659

[6] Zajenkowski M Jonason PK Leniarska M Kozakiewicz Z. Who complies with COVID-19 restrictions? The role of dark triad traits, collective narcissism, and health beliefs in predicting adherence to social distancing. J Soc Psychol. (2020) 160:639–49.

[7] Spoorthy MS Pratapa SK Mahant S. Mental health problems faced by healthcare workers due to the COVID-19 pandemic–A review. Asian J Psychiatr. (2020) 51:102119. 10.1016/j.ajp.2020.102119 32339895 PMC7175897

[8] Kowalska ME Pacian A. Burnout in medical personnel during the COVID-19 pandemic–a growing concern. Health Psychol Rep. (2020) 9:359–70.

[9] Fegert JM Vitiello B Plener PL Clemens V. Challenges and burden of the coronavirus 2019 (COVID-19) pandemic for child and adolescent mental health: A narrative review highlighting clinical and research needs in the acute phase and the long return to normality. Child Adolesc Psychiatry Ment Health. (2020) 14:1–11. 10.1186/s13034-020-00329-3 32419840 PMC7216870

[10] Ministerstwo Zdrowia (2021). Narodowy Program Ochrony Zdrowia Psychicznego 2017-2022. Available online at: https://www.gov.pl/web/zdrowie/narodowy-program-ochrony-zdrowia-psychicznego-na-lata-2017-2022 (accessed October 20, 2024).

[11] Rzecznik Praw Obywatelskich. Ochrona zdrowia psychicznego w Polsce – raport 2021. (2021). Available online at: https://www.rpo.gov.pl (accessed October 20, 2024).

[12] Kucharczyk M. Telepsychiatria: Rewolucja czy konieczność? Psychiatria Polska. (2021) 55:481–489.34460876

[13] Jedrychowska B Malesza M. Private psychiatric services in Poland in the context of public health care limitations during COVID-19 pandemic. Psychiatr Pol. (2020) 54:679–90.

[14] Płużański K Rzepa A. The rising role of private mental health care services in post-pandemic Poland. J Public Health Policy. (2021) 42:188–202.

[15] WHO. Mental Health and Psychosocial Considerations during the COVID-19 Outbreak. (2021). Available online at: https://www.who.int/docs/default-source/coronaviruse/mental-health-considerations.pdf (accessed October 20, 2024).

[16] Jakubczyk A Wojnar M. Psychiatric disorders in Poland during the COVID-19 pandemic: insights from early clinical practice and review of the literature. Postepy Psychiatrii i Neurologii. (2021) 30:51–60.

[17] Fundacja Dajemy Dzieciom Siłe. Pandemia a zdrowie psychiczne dzieci i młodzieży: raport z badań. (2020). Available online at: https://fdds.pl (accessed October 20, 2024).

[18] Salari N Hosseinian-Far A Jalali R Vaisi-Raygani A Khaledi-Paveh B. Prevalence of stress, anxiety, depression among the general population during the COVID-19 pandemic: a systematic review and meta-analysis. Global Health. (2020) 16:57. 10.1186/s12992-020-00589-w 32631403 PMC7338126

[19] Fundacja IGA. Praca zdalna a zdrowie psychiczne podczas pandemii COVID-19: Raport z badań. (2021). Available online at: https://iga.org.pl

[20] Ministerstwo Zdrowia. Kampania społeczna: Twoje zdrowie psychiczne ma znaczenie. (2021). Available online at: https://www.gov.pl/web/zdrowie/twoje-zdrowie-psychiczne-ma-znaczenie (accessed October 20, 2024).

[21] UNICEF. Mental health impact of COVID-19 on children and young people. (2021). Available online at: https://www.unicef.org/reports (accessed October 20, 2024).

[22] World Health Organization. Czechia: Community-based mental health services – a lifeline during COVID-19. (2021). Available online at: https://www.who.int/europe/publications/m/item/czechia-community-based-mental-health-services-a-lifeline-during-covid-19-(2021) (accessed October 20, 2024).

[23] Sani G Janiri D Di Nicola M Janiri L Ferracuti S Kotzalidis GD. During the first wave of the COVID-19 pandemic, mental health services in Europe: Results from the EPA Ambassadors survey and implications for clinical practice. Eur Psychiat. (2020) 63:e41. 34103102 10.1192/j.eurpsy.2021.2215 PMC8314055

[24] The impact of COVID-19 on mental health services in Eastern Europe: Challenges and opportunities. BMC Health Services Res. 23:9529.

[25] World Health Organization. Mental Health Atlas 2020. (2021). Available online at: https://www.who.int/publications/i/item/9789240036703 (accessed October 20, 2024).

[26] Gavidia M Smith M. Telemedicine and mental health care: Bridging gaps during and after the pandemic. BMJ. (2022) 380:e072398.38621153

[27] Moreno C Wykes T Galderisi S Nordentoft M Crossley N Jones N . How mental health care should change as a consequence of the COVID-19 pandemic. Lancet Psychiat. (2020) 7:813–824. 10.1016/S2215-0366(20)30307-2 32682460 PMC7365642

[28] Shore JH Schneck CD Mishkind MC. Telepsychiatry and the transformation of mental health care. Psychiatr Serv. (2020) 71:747–750.

[29] Kiejna A Piotrowski P Rymaszewska J. Telepsychiatria – skuteczność i ograniczenia w porównaniu do wizyt osobistych. Psychiatria. (2021) 18:57–66.

[30] Humer E Stippl P Pieh C Probst T. Psychoterapia online: skuteczność w porównaniu z terapia twarza w twarz na podstawie badań austriackich psychoterapeutów. Psychoterapia. (2021) 18:33–45.

[31] Nowak A Kowalczyk R Wójcik M. Ocena usług zdalnych i satysfakcja pacjentów – badanie jakościowe. ResearchGate. (2023). Available online at: https://www.researchgate.net/publication/373029815_Evaluation_of_remote_services_and_patient_satisfaction (accessed February 22, 2025).

[32] Medicover. Pandemia nasiliła problemy zdrowotne i absencje wśród pracowników. Zyskuja firmy inwestujace w prywatna opieke medyczna. Pobrano z (2021). Available online at: https://biuroprasowe.medicover.pl/173350-pandemia-nasilila-problemy-zdrowotne-i-absencje-wsrod-pracownikow-zyskuja-firmy-inwestujace-w-prywatna-opieke-medyczna (accessed October 20, 2024).

